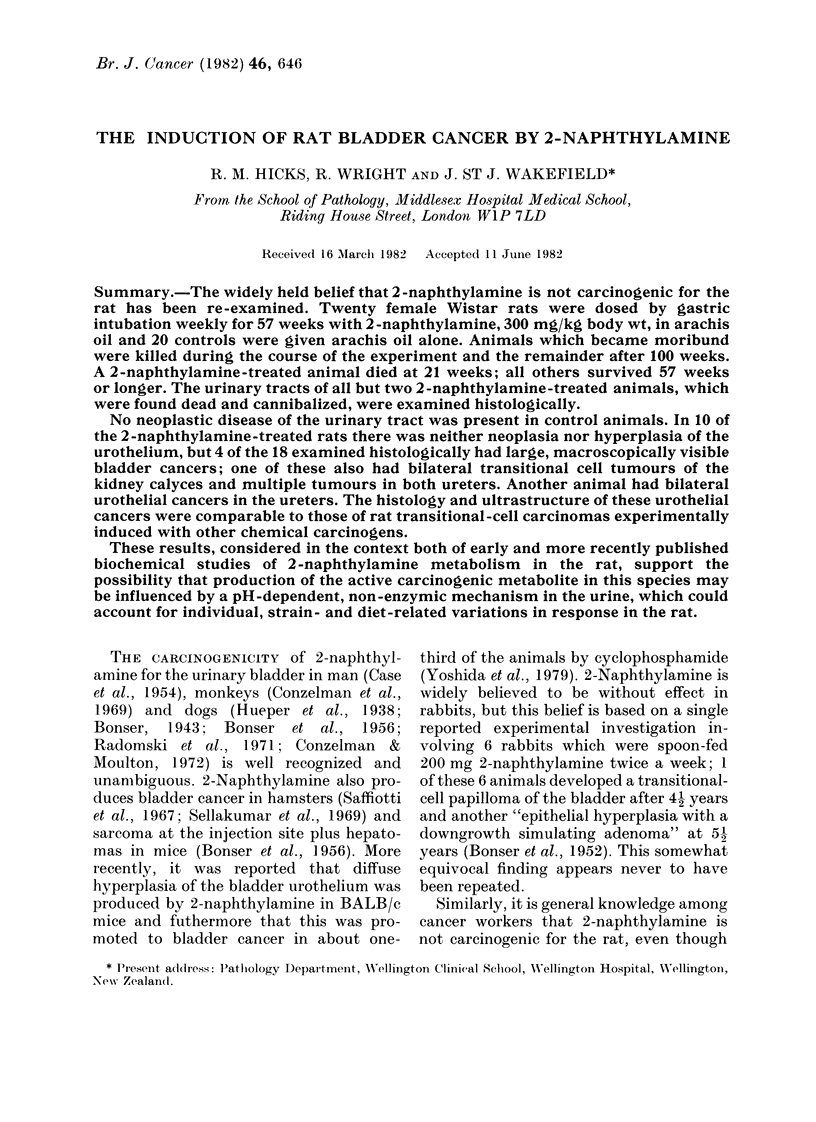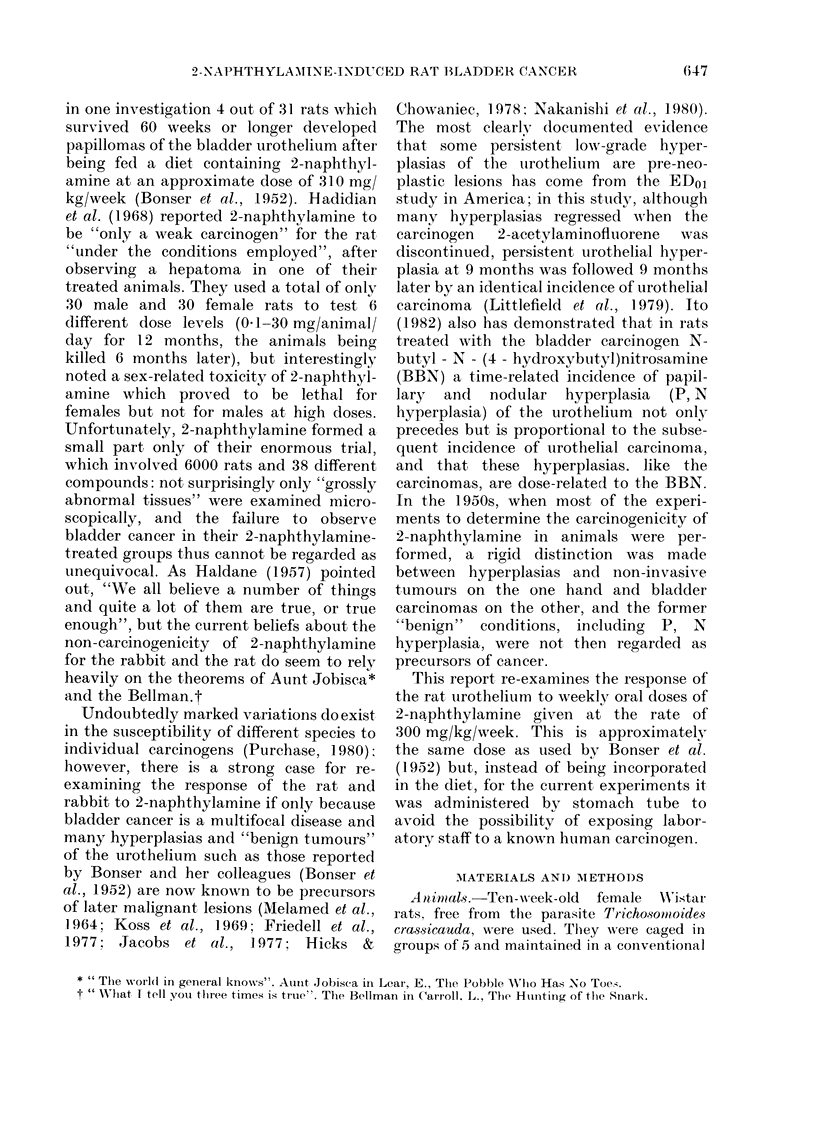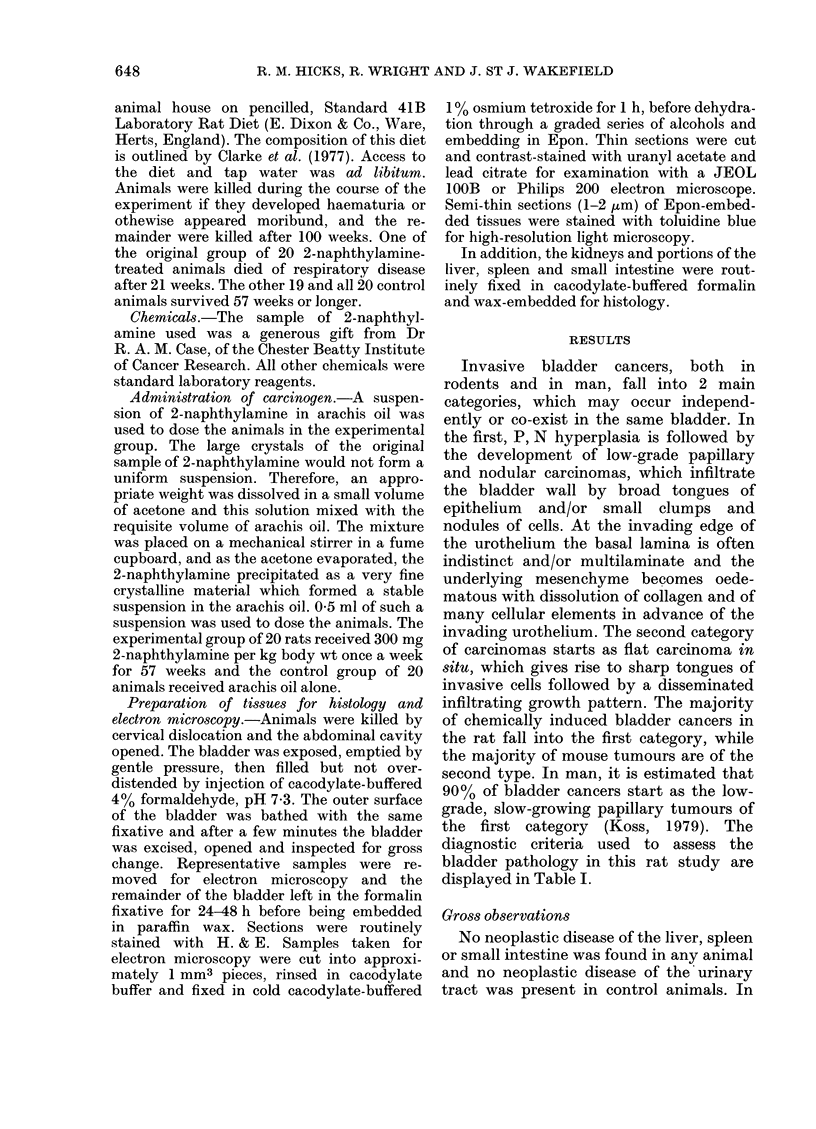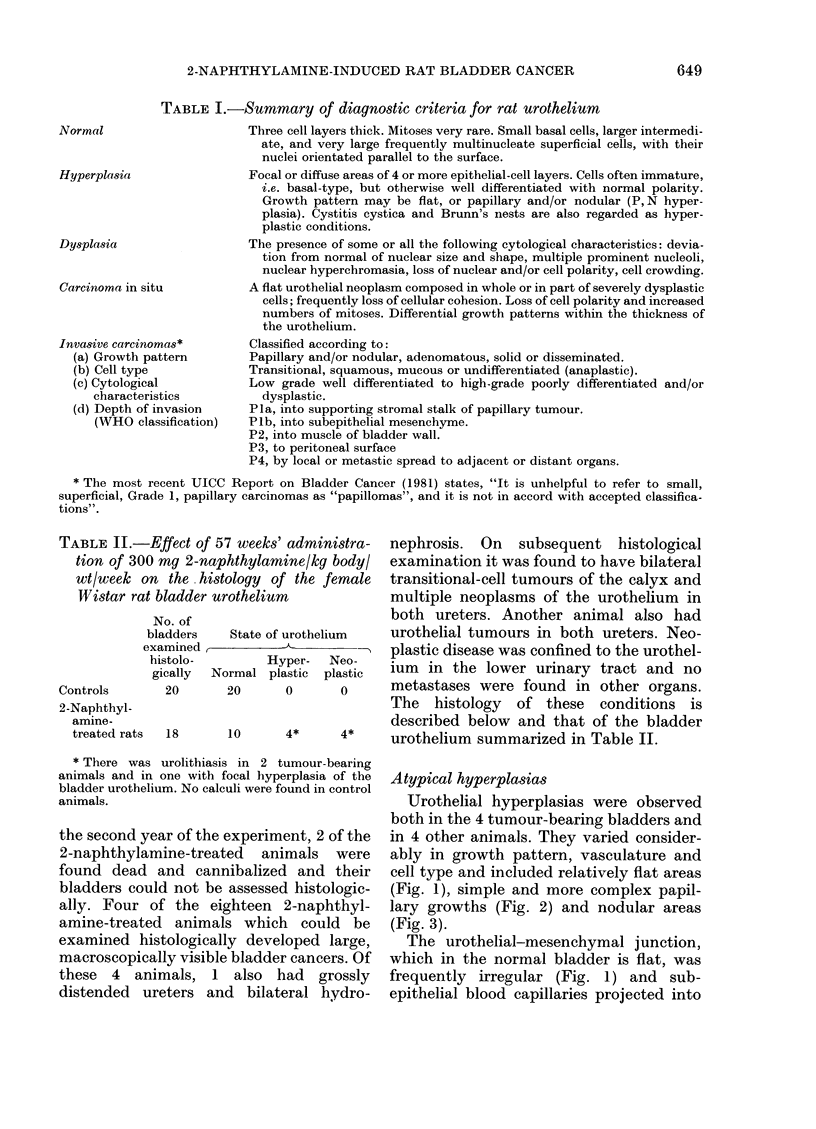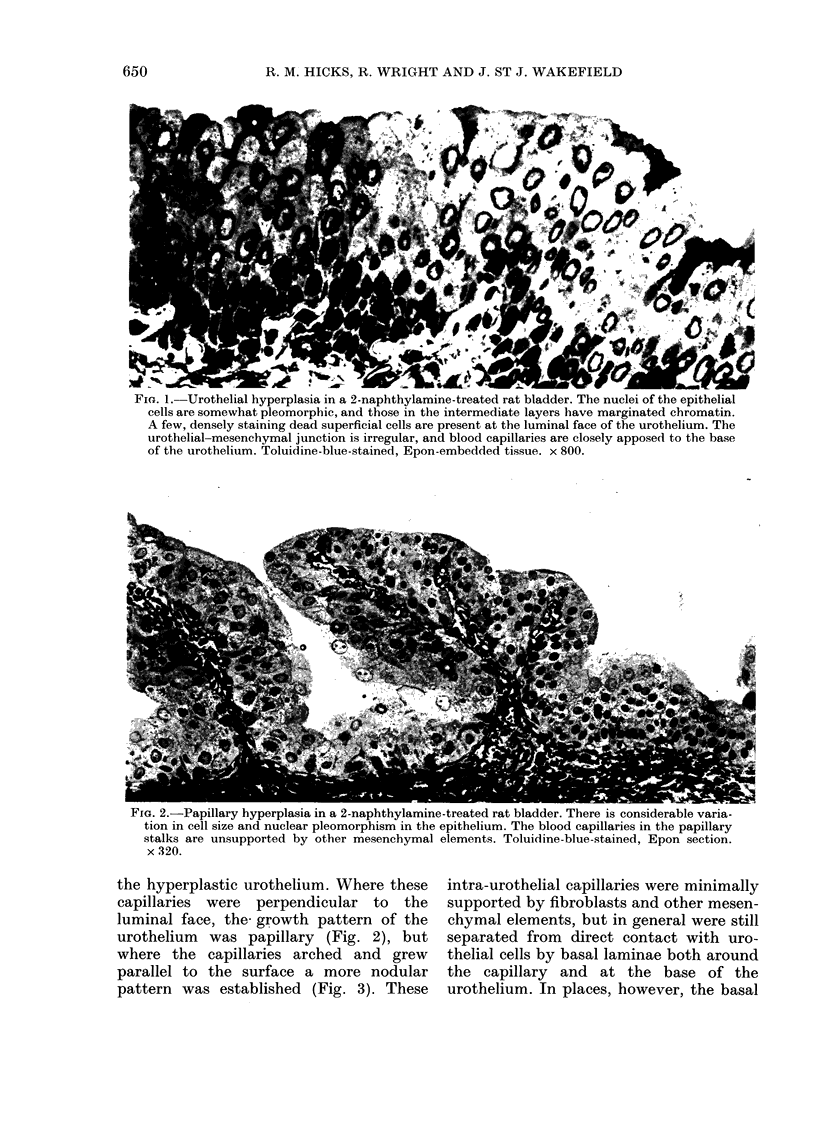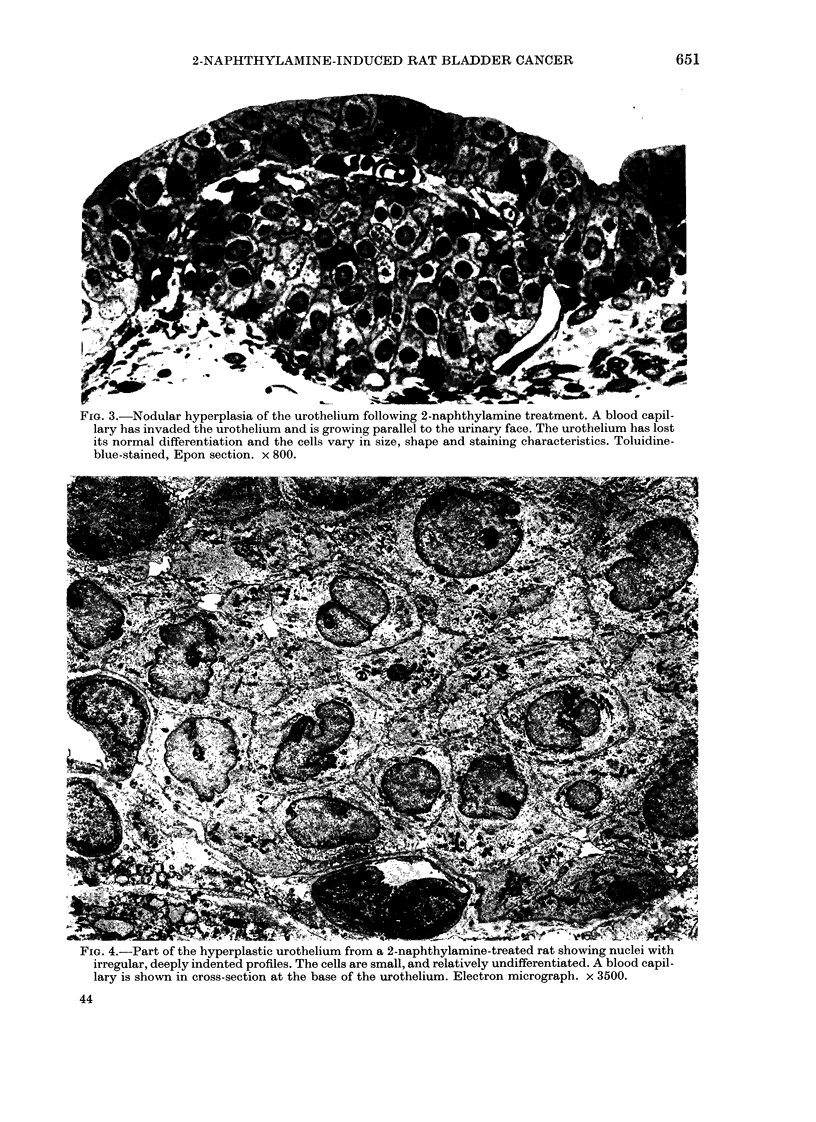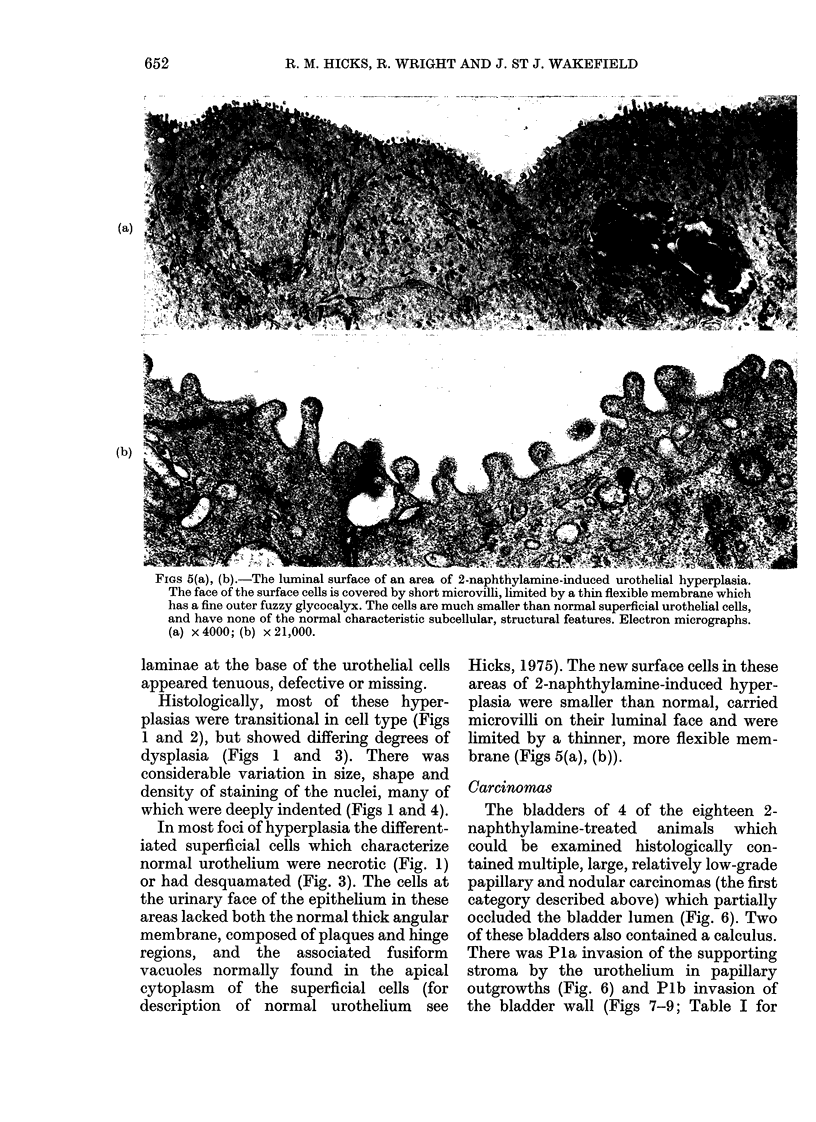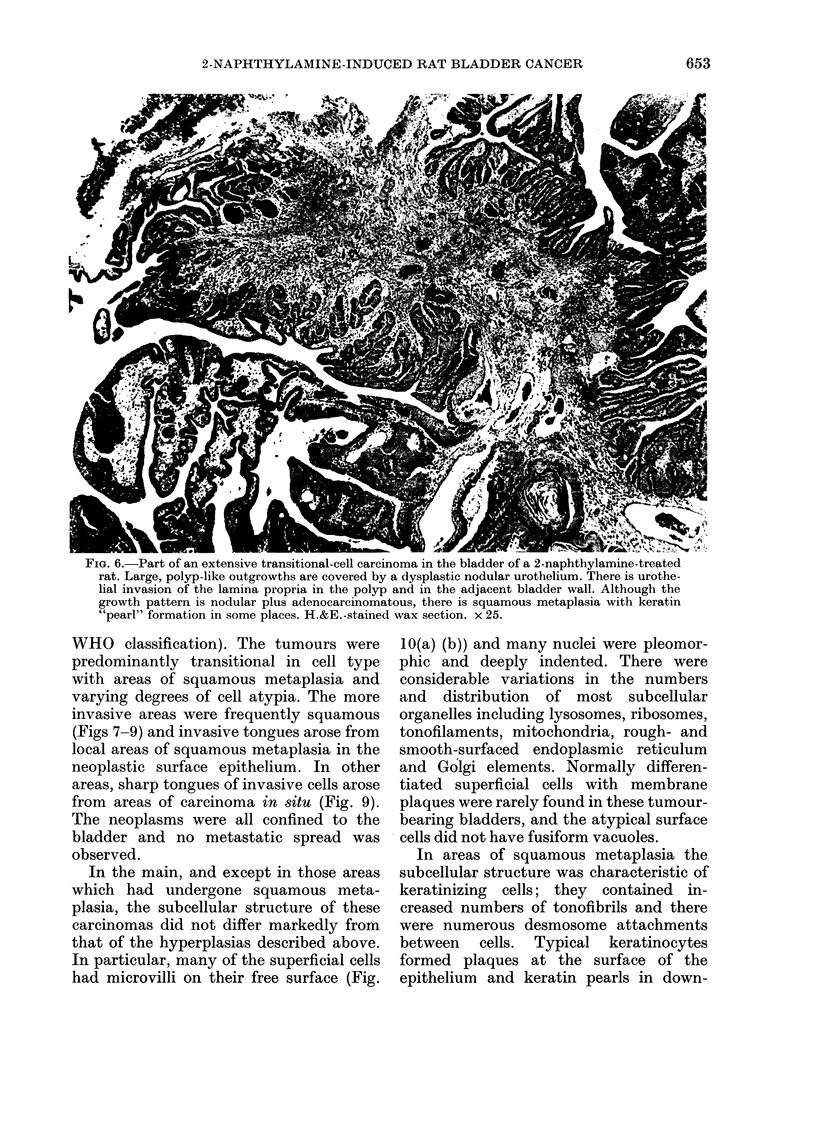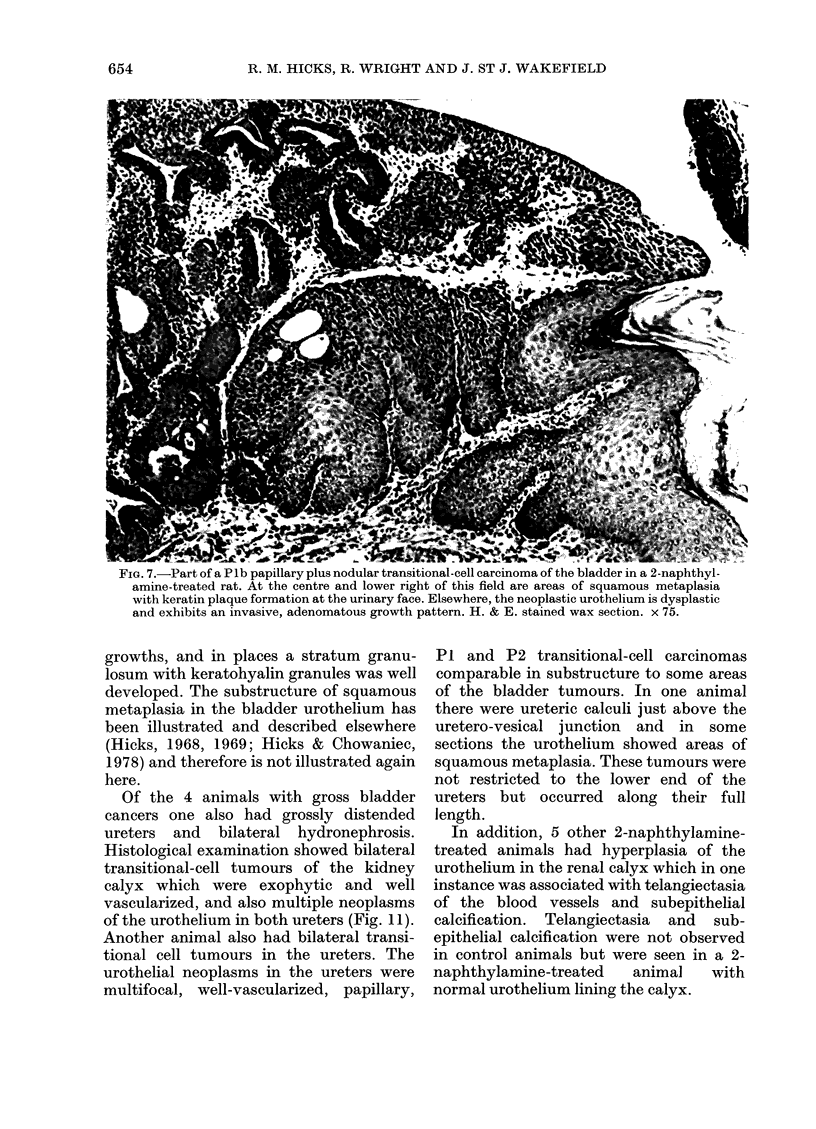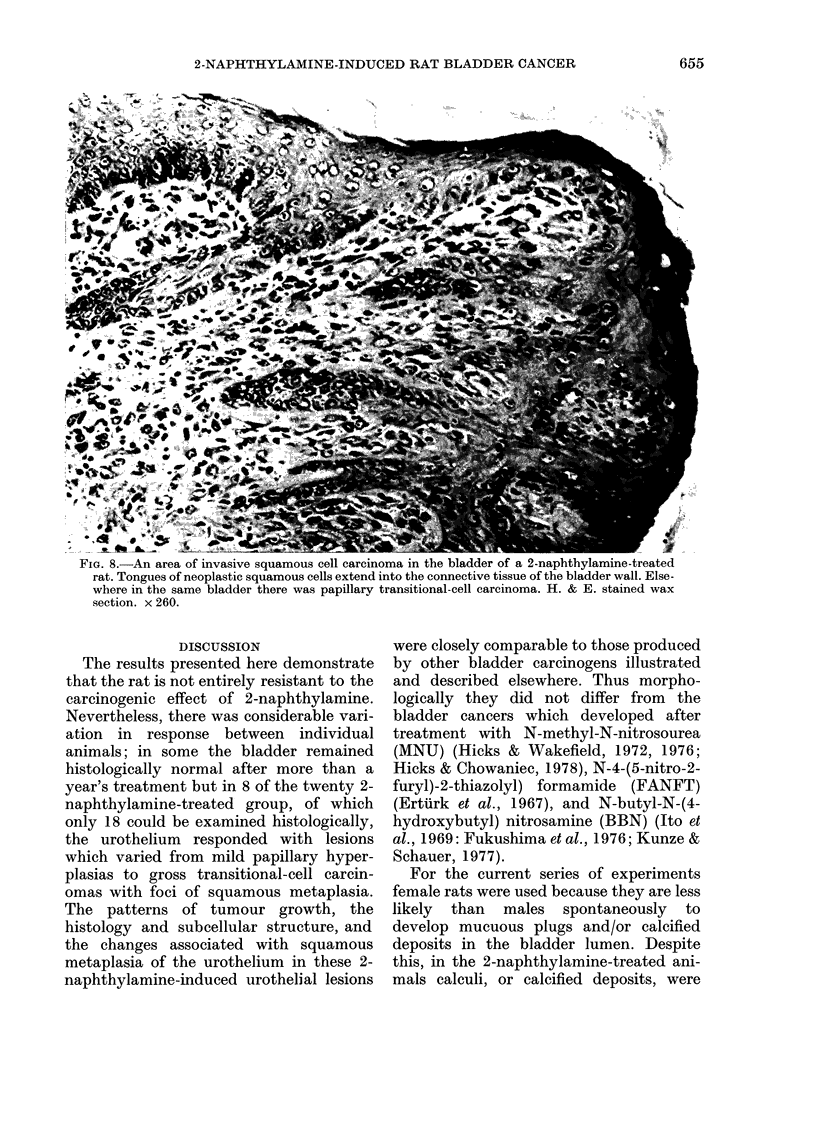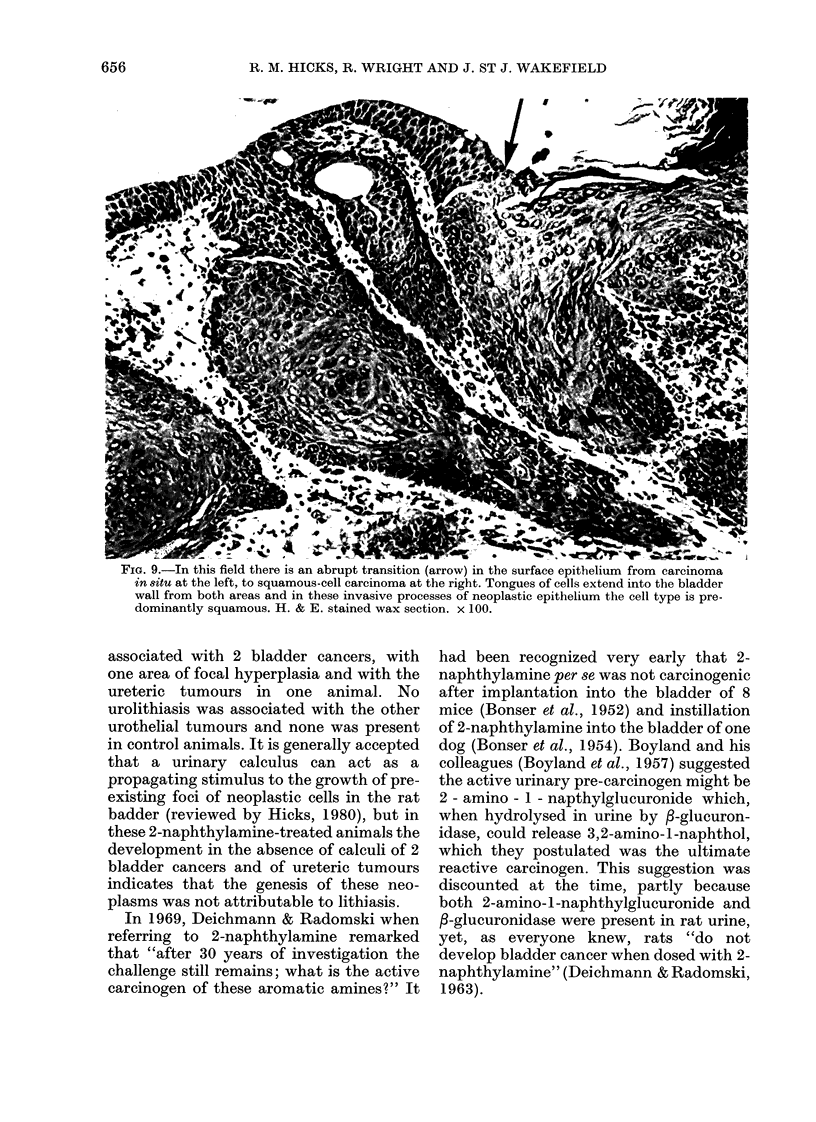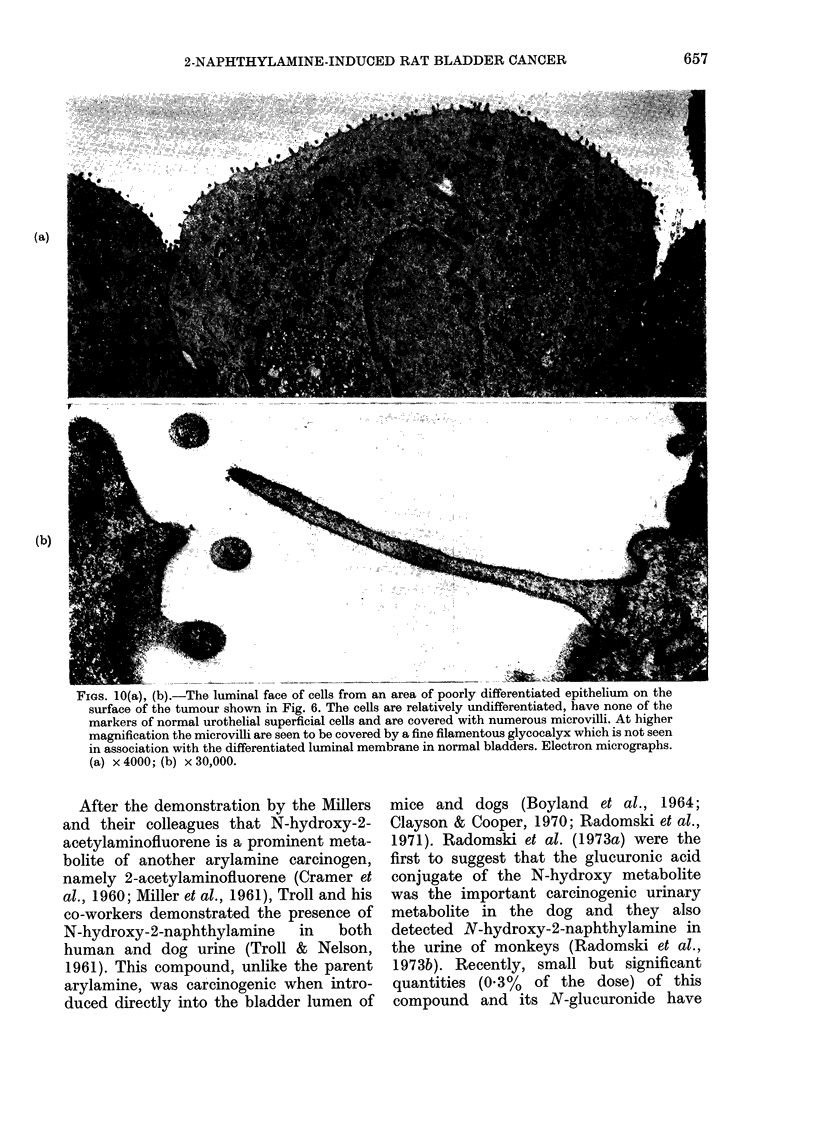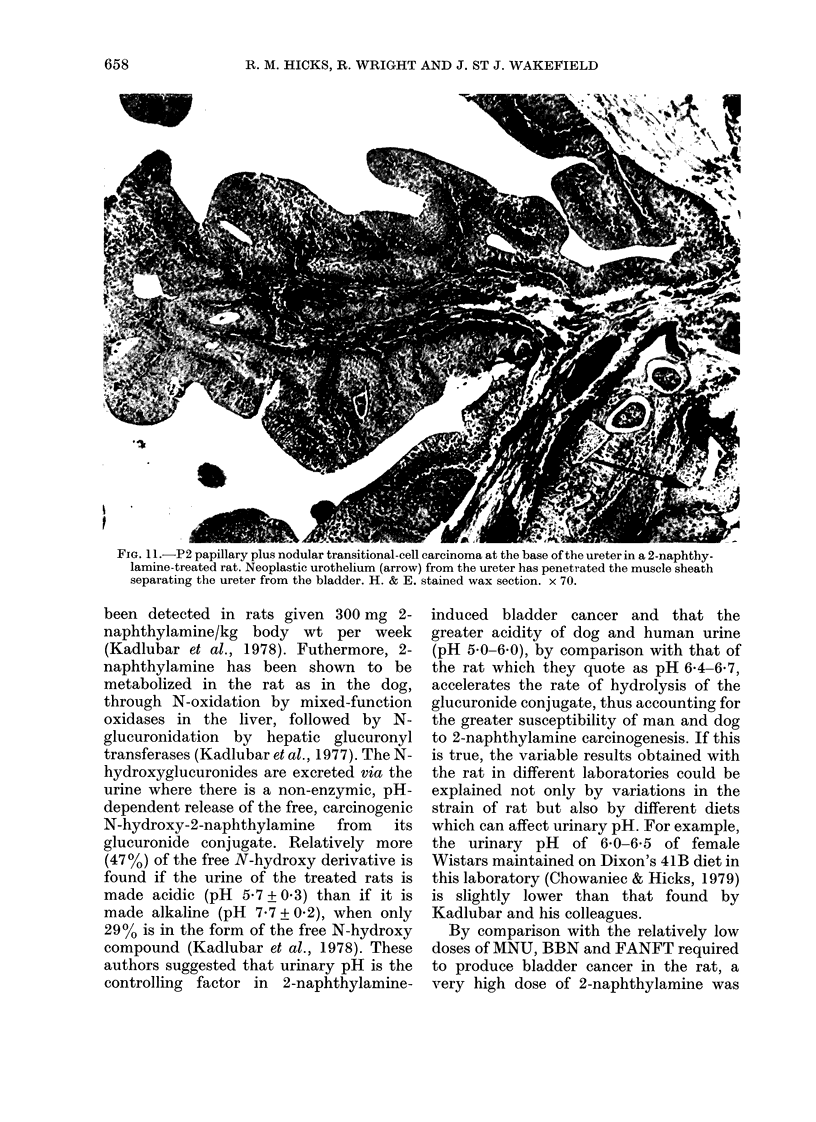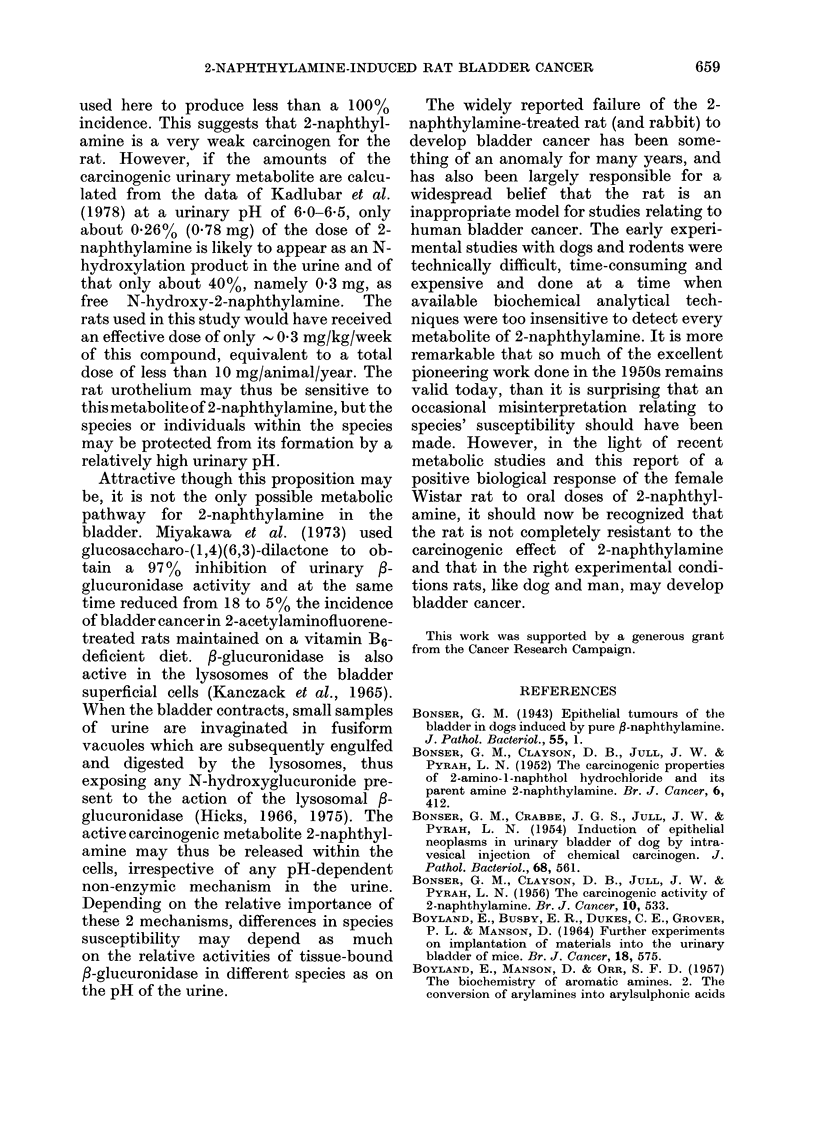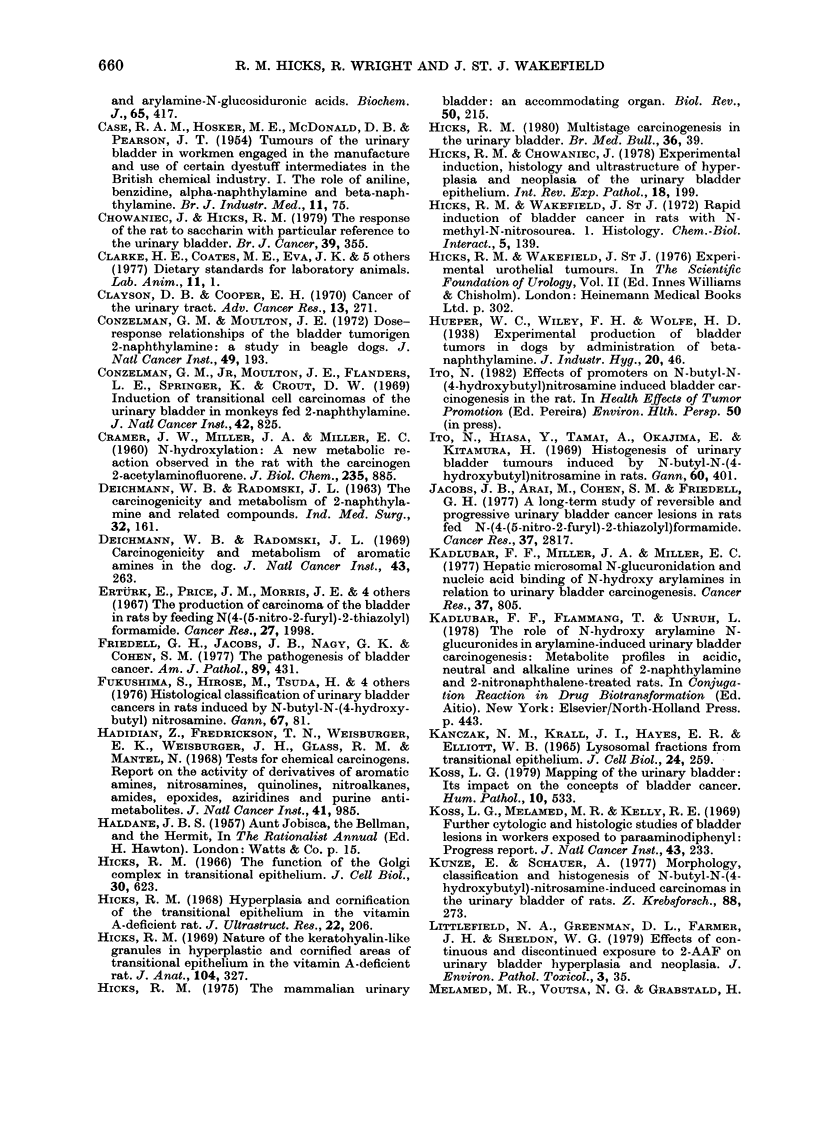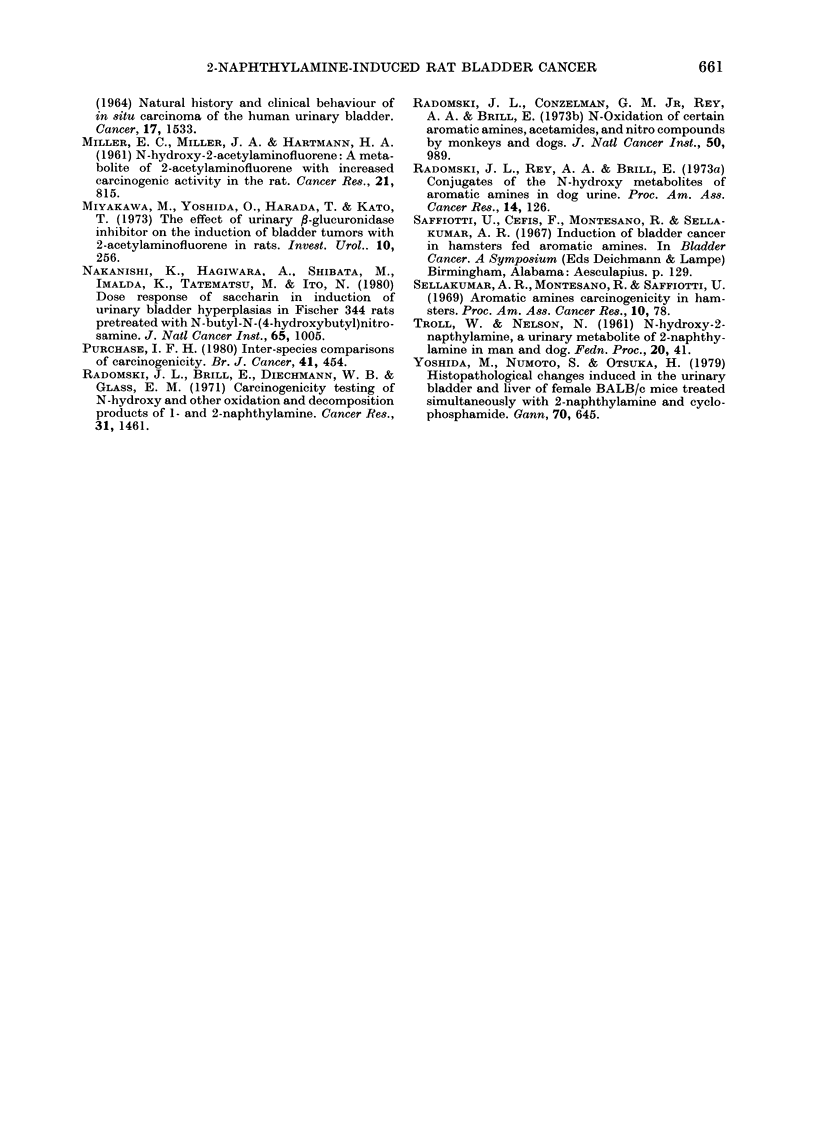# The induction of rat bladder cancer by 2-naphthylamine

**DOI:** 10.1038/bjc.1982.250

**Published:** 1982-10

**Authors:** R. M. Hicks, R. Wright, J. St J. Wakefield

## Abstract

**Images:**


*Br. J. Cancer* (1982) **46**, 646

### THE INDUCTION OF RAT BLADDER CANCER BY 2-NAPHTHYLAMINE

R. M. HICKS, R. WRIGHT AND J. ST J. WAKEFIELD*

*From the School of Pathology, Middlesex Hospital Medical School, Riding House Street, London W1P 7LD*

Received 16 March 1982 Accepted 11 June 1982

**Summary.—The widely held belief that 2-naphthylamine is not carcinogenic for the rat has been re-examined. Twenty female Wistar rats were dosed by gastric intubation weekly for 57 weeks with 2-naphthylamine, 300 mg/kg body wt, in arachis oil and 20 controls were given arachis oil alone. Animals which became moribund were killed during the course of the experiment and the remainder after 100 weeks. A 2-naphthylamine-treated animal died at 21 weeks; all others survived 57 weeks or longer. The urinary tracts of all but two 2-naphthylamine-treated animals, which were found dead and cannibalized, were examined histologically.**

**No neoplastic disease of the urinary tract was present in control animals. In 10 of the 2-naphthylamine-treated rats there was neither neoplasia nor hyperplasia of the urothelium, but 4 of the 18 examined histologically had large, macroscopically visible bladder cancers; one of these also had bilateral transitional cell tumours of the kidney calyces and multiple tumours in both ureters. Another animal had bilateral urothelial cancers in the ureters. The histology and ultrastructure of these urothelial cancers were comparable to those of rat transitional-cell carcinomas experimentally induced with other chemical carcinogens.**

**These results, considered in the context both of early and more recently published biochemical studies of 2-naphthylamine metabolism in the rat, support the possibility that production of the active carcinogenic metabolite in this species may be influenced by a pH-dependent, non-enzymic mechanism in the urine, which could account for individual, strain- and diet-related variations in response in the rat.**

THE CARCINOGENICITY of 2-naphthylamine for the urinary bladder in man (Case *et al.*, 1954), monkeys (Conzelman *et al.*, 1969) and dogs (Hueper *et al.*, 1938; Bonser, 1943; Bonser *et al.*, 1956; Radomski *et al.*, 1971; Conzelman & Moulton, 1972) is well recognized and unambiguous. 2-Naphthylamine also produces bladder cancer in hamsters (Saffiotti *et al.*, 1967; Sellakumar *et al.*, 1969) and sarcoma at the injection site plus hepatomas in mice (Bonser *et al.*, 1956). More recently, it was reported that diffuse hyperplasia of the bladder urothelium was produced by 2-naphthylamine in BALB/c mice and futhermore that this was promoted to bladder cancer in about one-third of the animals by cyclophosphamide (Yoshida *et al.*, 1979). 2-Naphthylamine is widely believed to be without effect in rabbits, but this belief is based on a single reported experimental investigation involving 6 rabbits which were spoon-fed 200 mg 2-naphthylamine twice a week; 1 of these 6 animals developed a transitional-cell papilloma of the bladder after $4\frac{1}{2}$ years and another "epithelial hyperplasia with a downgrowth simulating adenoma" at $5\frac{1}{2}$ years (Bonser *et al.*, 1952). This somewhat equivocal finding appears never to have been repeated.

Similarly, it is general knowledge among cancer workers that 2-naphthylamine is not carcinogenic for the rat, even though

* Present address: Pathology Department, Wellington Clinical School, Wellington Hospital, Wellington, New Zealand.

in one investigation 4 out of 31 rats which survived 60 weeks or longer developed papillomas of the bladder urothelium after being fed a diet containing 2-naphthylamine at an approximate dose of 310 mg/kg/week (Bonser *et al.*, 1952). Hadidian *et al.* (1968) reported 2-naphthylamine to be "only a weak carcinogen" for the rat "under the conditions employed", after observing a hepatoma in one of their treated animals. They used a total of only 30 male and 30 female rats to test 6 different dose levels (0·1–30 mg/animal/day for 12 months, the animals being killed 6 months later), but interestingly noted a sex-related toxicity of 2-naphthylamine which proved to be lethal for females but not for males at high doses. Unfortunately, 2-naphthylamine formed a small part only of their enormous trial, which involved 6000 rats and 38 different compounds: not surprisingly only "grossly abnormal tissues" were examined microscopically, and the failure to observe bladder cancer in their 2-naphthylamine-treated groups thus cannot be regarded as unequivocal. As Haldane (1957) pointed out, "We all believe a number of things and quite a lot of them are true, or true enough", but the current beliefs about the non-carcinogenicity of 2-naphthylamine for the rabbit and the rat do seem to rely heavily on the theorems of Aunt Jobisca* and the Bellman.†

Undoubtedly marked variations do exist in the susceptibility of different species to individual carcinogens (Purchase, 1980); however, there is a strong case for re-examining the response of the rat and rabbit to 2-naphthylamine if only because bladder cancer is a multifocal disease and many hyperplasias and "benign tumours" of the urothelium such as those reported by Bonser and her colleagues (Bonser *et al.*, 1952) are now known to be precursors of later malignant lesions (Melamed *et al.*, 1964; Koss *et al.*, 1969; Friedell *et al.*, 1977; Jacobs *et al.*, 1977; Hicks & Chowaniec, 1978; Nakanishi *et al.*, 1980). The most clearly documented evidence that some persistent low-grade hyperplasias of the urothelium are pre-neoplastic lesions has come from the $ED_{01}$ study in America; in this study, although many hyperplasias regressed when the carcinogen 2-acetylaminofluorene was discontinued, persistent urothelial hyperplasia at 9 months was followed 9 months later by an identical incidence of urothelial carcinoma (Littlefield *et al.*, 1979). Ito (1982) also has demonstrated that in rats treated with the bladder carcinogen N-butyl - N - (4 - hydroxybutyl)nitrosamine (BBN) a time-related incidence of papillary and nodular hyperplasia (P, N hyperplasia) of the urothelium not only precedes but is proportional to the subsequent incidence of urothelial carcinoma, and that these hyperplasias, like the carcinomas, are dose-related to the BBN. In the 1950s, when most of the experiments to determine the carcinogenicity of 2-naphthylamine in animals were performed, a rigid distinction was made between hyperplasias and non-invasive tumours on the one hand and bladder carcinomas on the other, and the former "benign" conditions, including P, N hyperplasia, were not then regarded as precursors of cancer.

This report re-examines the response of the rat urothelium to weekly oral doses of 2-naphthylamine given at the rate of 300 mg/kg/week. This is approximately the same dose as used by Bonser *et al.* (1952) but, instead of being incorporated in the diet, for the current experiments it was administered by stomach tube to avoid the possibility of exposing laboratory staff to a known human carcinogen.

### MATERIALS AND METHODS

*Animals.*—Ten-week-old female Wistar rats, free from the parasite *Trichosomoides crassicauda*, were used. They were caged in groups of 5 and maintained in a conventional

* " The world in general knows". Aunt Jobisca in Lear, E., The Pobble Who Has No Toes.

† " What I tell you three times is true". The Bellman in Carroll, L., The Hunting of the Snark.

animal house on pencilled, Standard 41B Laboratory Rat Diet (E. Dixon & Co., Ware, Herts, England). The composition of this diet is outlined by Clarke *et al.* (1977). Access to the diet and tap water was *ad libitum*. Animals were killed during the course of the experiment if they developed haematuria or othewise appeared moribund, and the remainder were killed after 100 weeks. One of the original group of 20 2-naphthylamine-treated animals died of respiratory disease after 21 weeks. The other 19 and all 20 control animals survived 57 weeks or longer.

*Chemicals.*—The sample of 2-naphthylamine used was a generous gift from Dr R. A. M. Case, of the Chester Beatty Institute of Cancer Research. All other chemicals were standard laboratory reagents.

*Administration of carcinogen.*—A suspension of 2-naphthylamine in arachis oil was used to dose the animals in the experimental group. The large crystals of the original sample of 2-naphthylamine would not form a uniform suspension. Therefore, an appropriate weight was dissolved in a small volume of acetone and this solution mixed with the requisite volume of arachis oil. The mixture was placed on a mechanical stirrer in a fume cupboard, and as the acetone evaporated, the 2-naphthylamine precipitated as a very fine crystalline material which formed a stable suspension in the arachis oil. 0·5 ml of such a suspension was used to dose the animals. The experimental group of 20 rats received 300 mg 2-naphthylamine per kg body wt once a week for 57 weeks and the control group of 20 animals received arachis oil alone.

*Preparation of tissues for histology and electron microscopy.*—Animals were killed by cervical dislocation and the abdominal cavity opened. The bladder was exposed, emptied by gentle pressure, then filled but not overdistended by injection of cacodylate-buffered 4% formaldehyde, pH 7·3. The outer surface of the bladder was bathed with the same fixative and after a few minutes the bladder was excised, opened and inspected for gross change. Representative samples were removed for electron microscopy and the remainder of the bladder left in the formalin fixative for 24–48 h before being embedded in paraffin wax. Sections were routinely stained with H. & E. Samples taken for electron microscopy were cut into approximately 1 $mm^3$ pieces, rinsed in cacodylate buffer and fixed in cold cacodylate-buffered 1% osmium tetroxide for 1 h, before dehydration through a graded series of alcohols and embedding in Epon. Thin sections were cut and contrast-stained with uranyl acetate and lead citrate for examination with a JEOL 100B or Philips 200 electron microscope. Semi-thin sections (1–2 $\mu$m) of Epon-embedded tissues were stained with toluidine blue for high-resolution light microscopy.

In addition, the kidneys and portions of the liver, spleen and small intestine were routinely fixed in cacodylate-buffered formalin and wax-embedded for histology.

### RESULTS

Invasive bladder cancers, both in rodents and in man, fall into 2 main categories, which may occur independently or co-exist in the same bladder. In the first, P, N hyperplasia is followed by the development of low-grade papillary and nodular carcinomas, which infiltrate the bladder wall by broad tongues of epithelium and/or small clumps and nodules of cells. At the invading edge of the urothelium the basal lamina is often indistinct and/or multilaminate and the underlying mesenchyme becomes oedematous with dissolution of collagen and of many cellular elements in advance of the invading urothelium. The second category of carcinomas starts as flat carcinoma *in situ*, which gives rise to sharp tongues of invasive cells followed by a disseminated infiltrating growth pattern. The majority of chemically induced bladder cancers in the rat fall into the first category, while the majority of mouse tumours are of the second type. In man, it is estimated that 90% of bladder cancers start as the low-grade, slow-growing papillary tumours of the first category (Koss, 1979). The diagnostic criteria used to assess the bladder pathology in this rat study are displayed in Table I.

### *Gross observations*

No neoplastic disease of the liver, spleen or small intestine was found in any animal and no neoplastic disease of the urinary tract was present in control animals. In

TABLE I.—*Summary of diagnostic criteria for rat urothelium*

| | |
|---|---|
| *Normal* | Three cell layers thick. Mitoses very rare. Small basal cells, larger intermediate, and very large frequently multinucleate superficial cells, with their nuclei orientated parallel to the surface. |
| *Hyperplasia* | Focal or diffuse areas of 4 or more epithelial-cell layers. Cells often immature, *i.e.* basal-type, but otherwise well differentiated with normal polarity. Growth pattern may be flat, or papillary and/or nodular (P, N hyperplasia). Cystitis cystica and Brunn's nests are also regarded as hyperplastic conditions. |
| *Dysplasia* | The presence of some or all the following cytological characteristics: deviation from normal of nuclear size and shape, multiple prominent nucleoli, nuclear hyperchromasia, loss of nuclear and/or cell polarity, cell crowding. |
| *Carcinoma* in situ | A flat urothelial neoplasm composed in whole or in part of severely dysplastic cells; frequently loss of cellular cohesion. Loss of cell polarity and increased numbers of mitoses. Differential growth patterns within the thickness of the urothelium. |
| *Invasive carcinomas** | Classified according to: |
| (a) Growth pattern | Papillary and/or nodular, adenomatous, solid or disseminated. |
| (b) Cell type | Transitional, squamous, mucous or undifferentiated (anaplastic). |
| (c) Cytological characteristics | Low grade well differentiated to high-grade poorly differentiated and/or dysplastic. |
| (d) Depth of invasion (WHO classification) | P1a, into supporting stromal stalk of papillary tumour.<br>P1b, into subepithelial mesenchyme.<br>P2, into muscle of bladder wall.<br>P3, to peritoneal surface<br>P4, by local or metastic spread to adjacent or distant organs. |

* The most recent UICC Report on Bladder Cancer (1981) states, "It is unhelpful to refer to small, superficial, Grade 1, papillary carcinomas as "papillomas", and it is not in accord with accepted classifications".

TABLE II.—*Effect of* 57 *weeks' administration of* 300 *mg 2-naphthylamine/kg body/wt/week on the histology of the female Wistar rat bladder urothelium*

| | No. of bladders examined histologically | State of urothelium | | |
|---|---|---|---|---|
| | | Normal | Hyperplastic | Neoplastic |
| Controls | 20 | 20 | 0 | 0 |
| 2-Naphthylamine-treated rats | 18 | 10 | 4* | 4* |

* There was urolithiasis in 2 tumour-bearing animals and in one with focal hyperplasia of the bladder urothelium. No calculi were found in control animals.

the second year of the experiment, 2 of the 2-naphthylamine-treated animals were found dead and cannibalized and their bladders could not be assessed histologically. Four of the eighteen 2-naphthylamine-treated animals which could be examined histologically developed large, macroscopically visible bladder cancers. Of these 4 animals, 1 also had grossly distended ureters and bilateral hydronephrosis. On subsequent histological examination it was found to have bilateral transitional-cell tumours of the calyx and multiple neoplasms of the urothelium in both ureters. Another animal also had urothelial tumours in both ureters. Neoplastic disease was confined to the urothelium in the lower urinary tract and no metastases were found in other organs. The histology of these conditions is described below and that of the bladder urothelium summarized in Table II.

### *Atypical hyperplasias*

Urothelial hyperplasias were observed both in the 4 tumour-bearing bladders and in 4 other animals. They varied considerably in growth pattern, vasculature and cell type and included relatively flat areas (Fig. 1), simple and more complex papillary growths (Fig. 2) and nodular areas (Fig. 3).

The urothelial–mesenchymal junction, which in the normal bladder is flat, was frequently irregular (Fig. 1) and subepithelial blood capillaries projected into

FIG. 1.—Urothelial hyperplasia in a 2-naphthylamine-treated rat bladder. The nuclei of the epithelial cells are somewhat pleomorphic, and those in the intermediate layers have marginated chromatin. A few, densely staining dead superficial cells are present at the luminal face of the urothelium. The urothelial–mesenchymal junction is irregular, and blood capillaries are closely apposed to the base of the urothelium. Toluidine-blue-stained, Epon-embedded tissue. × 800.

FIG. 2.—Papillary hyperplasia in a 2-naphthylamine-treated rat bladder. There is considerable variation in cell size and nuclear pleomorphism in the epithelium. The blood capillaries in the papillary stalks are unsupported by other mesenchymal elements. Toluidine-blue-stained, Epon section. × 320.

the hyperplastic urothelium. Where these capillaries were perpendicular to the luminal face, the growth pattern of the urothelium was papillary (Fig. 2), but where the capillaries arched and grew parallel to the surface a more nodular pattern was established (Fig. 3). These intra-urothelial capillaries were minimally supported by fibroblasts and other mesenchymal elements, but in general were still separated from direct contact with urothelial cells by basal laminae both around the capillary and at the base of the urothelium. In places, however, the basal

FIG. 3.—Nodular hyperplasia of the urothelium following 2-naphthylamine treatment. A blood capillary has invaded the urothelium and is growing parallel to the urinary face. The urothelium has lost its normal differentiation and the cells vary in size, shape and staining characteristics. Toluidine-blue-stained, Epon section. × 800.

FIG. 4.—Part of the hyperplastic urothelium from a 2-naphthylamine-treated rat showing nuclei with irregular, deeply indented profiles. The cells are small, and relatively undifferentiated. A blood capillary is shown in cross-section at the base of the urothelium. Electron micrograph. × 3500.

FIGS 5(a), (b).—The luminal surface of an area of 2-naphthylamine-induced urothelial hyperplasia. The face of the surface cells is covered by short microvilli, limited by a thin flexible membrane which has a fine outer fuzzy glycocalyx. The cells are much smaller than normal superficial urothelial cells, and have none of the normal characteristic subcellular, structural features. Electron micrographs. (a) ×4000; (b) ×21,000.

laminae at the base of the urothelial cells appeared tenuous, defective or missing.

Histologically, most of these hyperplasias were transitional in cell type (Figs 1 and 2), but showed differing degrees of dysplasia (Figs 1 and 3). There was considerable variation in size, shape and density of staining of the nuclei, many of which were deeply indented (Figs 1 and 4).

In most foci of hyperplasia the differentiated superficial cells which characterize normal urothelium were necrotic (Fig. 1) or had desquamated (Fig. 3). The cells at the urinary face of the epithelium in these areas lacked both the normal thick angular membrane, composed of plaques and hinge regions, and the associated fusiform vacuoles normally found in the apical cytoplasm of the superficial cells (for description of normal urothelium see Hicks, 1975). The new surface cells in these areas of 2-naphthylamine-induced hyperplasia were smaller than normal, carried microvilli on their luminal face and were limited by a thinner, more flexible membrane (Figs 5(a), (b)).

*Carcinomas*

The bladders of 4 of the eighteen 2-naphthylamine-treated animals which could be examined histologically contained multiple, large, relatively low-grade papillary and nodular carcinomas (the first category described above) which partially occluded the bladder lumen (Fig. 6). Two of these bladders also contained a calculus. There was P1a invasion of the supporting stroma by the urothelium in papillary outgrowths (Fig. 6) and P1b invasion of the bladder wall (Figs 7–9; Table I for

FIG. 6.—Part of an extensive transitional-cell carcinoma in the bladder of a 2-naphthylamine-treated rat. Large, polyp-like outgrowths are covered by a dysplastic nodular urothelium. There is urothelial invasion of the lamina propria in the polyp and in the adjacent bladder wall. Although the growth pattern is nodular plus adenocarcinomatous, there is squamous metaplasia with keratin "pearl" formation in some places. H.&E.-stained wax section. ×25.

WHO classification). The tumours were predominantly transitional in cell type with areas of squamous metaplasia and varying degrees of cell atypia. The more invasive areas were frequently squamous (Figs 7–9) and invasive tongues arose from local areas of squamous metaplasia in the neoplastic surface epithelium. In other areas, sharp tongues of invasive cells arose from areas of carcinoma *in situ* (Fig. 9). The neoplasms were all confined to the bladder and no metastatic spread was observed.

In the main, and except in those areas which had undergone squamous metaplasia, the subcellular structure of these carcinomas did not differ markedly from that of the hyperplasias described above. In particular, many of the superficial cells had microvilli on their free surface (Fig. 10(a) (b)) and many nuclei were pleomorphic and deeply indented. There were considerable variations in the numbers and distribution of most subcellular organelles including lysosomes, ribosomes, tonofilaments, mitochondria, rough- and smooth-surfaced endoplasmic reticulum and Golgi elements. Normally differentiated superficial cells with membrane plaques were rarely found in these tumour-bearing bladders, and the atypical surface cells did not have fusiform vacuoles.

In areas of squamous metaplasia the subcellular structure was characteristic of keratinizing cells; they contained increased numbers of tonofibrils and there were numerous desmosome attachments between cells. Typical keratinocytes formed plaques at the surface of the epithelium and keratin pearls in down-

FIG. 7.—Part of a P1b papillary plus nodular transitional-cell carcinoma of the bladder in a 2-naphthylamine-treated rat. At the centre and lower right of this field are areas of squamous metaplasia with keratin plaque formation at the urinary face. Elsewhere, the neoplastic urothelium is dysplastic and exhibits an invasive, adenomatous growth pattern. H. & E. stained wax section. × 75.

growths, and in places a stratum granulosum with keratohyalin granules was well developed. The substructure of squamous metaplasia in the bladder urothelium has been illustrated and described elsewhere (Hicks, 1968, 1969; Hicks & Chowaniec, 1978) and therefore is not illustrated again here.

Of the 4 animals with gross bladder cancers one also had grossly distended ureters and bilateral hydronephrosis. Histological examination showed bilateral transitional-cell tumours of the kidney calyx which were exophytic and well vascularized, and also multiple neoplasms of the urothelium in both ureters (Fig. 11). Another animal also had bilateral transitional cell tumours in the ureters. The urothelial neoplasms in the ureters were multifocal, well-vascularized, papillary, P1 and P2 transitional-cell carcinomas comparable in substructure to some areas of the bladder tumours. In one animal there were ureteric calculi just above the uretero-vesical junction and in some sections the urothelium showed areas of squamous metaplasia. These tumours were not restricted to the lower end of the ureters but occurred along their full length.

In addition, 5 other 2-naphthylamine-treated animals had hyperplasia of the urothelium in the renal calyx which in one instance was associated with telangiectasia of the blood vessels and subepithelial calcification. Telangiectasia and subepithelial calcification were not observed in control animals but were seen in a 2-naphthylamine-treated animal with normal urothelium lining the calyx.

FIG. 8.—An area of invasive squamous cell carcinoma in the bladder of a 2-naphthylamine-treated rat. Tongues of neoplastic squamous cells extend into the connective tissue of the bladder wall. Elsewhere in the same bladder there was papillary transitional-cell carcinoma. H. & E. stained wax section. × 260.

### DISCUSSION

The results presented here demonstrate that the rat is not entirely resistant to the carcinogenic effect of 2-naphthylamine. Nevertheless, there was considerable variation in response between individual animals; in some the bladder remained histologically normal after more than a year's treatment but in 8 of the twenty 2-naphthylamine-treated group, of which only 18 could be examined histologically, the urothelium responded with lesions which varied from mild papillary hyperplasias to gross transitional-cell carcinomas with foci of squamous metaplasia. The patterns of tumour growth, the histology and subcellular structure, and the changes associated with squamous metaplasia of the urothelium in these 2-naphthylamine-induced urothelial lesions were closely comparable to those produced by other bladder carcinogens illustrated and described elsewhere. Thus morphologically they did not differ from the bladder cancers which developed after treatment with N-methyl-N-nitrosourea (MNU) (Hicks & Wakefield, 1972, 1976; Hicks & Chowaniec, 1978), N-4-(5-nitro-2-furyl)-2-thiazolyl) formamide (FANFT) (Ertürk *et al.*, 1967), and N-butyl-N-(4-hydroxybutyl) nitrosamine (BBN) (Ito *et al.*, 1969: Fukushima *et al.*, 1976; Kunze & Schauer, 1977).

For the current series of experiments female rats were used because they are less likely than males spontaneously to develop mucuous plugs and/or calcified deposits in the bladder lumen. Despite this, in the 2-naphthylamine-treated animals calculi, or calcified deposits, were

FIG. 9.—In this field there is an abrupt transition (arrow) in the surface epithelium from carcinoma *in situ* at the left, to squamous-cell carcinoma at the right. Tongues of cells extend into the bladder wall from both areas and in these invasive processes of neoplastic epithelium the cell type is predominantly squamous. H. & E. stained wax section. × 100.

associated with 2 bladder cancers, with one area of focal hyperplasia and with the ureteric tumours in one animal. No urolithiasis was associated with the other urothelial tumours and none was present in control animals. It is generally accepted that a urinary calculus can act as a propagating stimulus to the growth of pre-existing foci of neoplastic cells in the rat badder (reviewed by Hicks, 1980), but in these 2-naphthylamine-treated animals the development in the absence of calculi of 2 bladder cancers and of ureteric tumours indicates that the genesis of these neoplasms was not attributable to lithiasis.

In 1969, Deichmann & Radomski when referring to 2-naphthylamine remarked that "after 30 years of investigation the challenge still remains; what is the active carcinogen of these aromatic amines?" It had been recognized very early that 2-naphthylamine *per se* was not carcinogenic after implantation into the bladder of 8 mice (Bonser *et al.*, 1952) and instillation of 2-naphthylamine into the bladder of one dog (Bonser *et al.*, 1954). Boyland and his colleagues (Boyland *et al.*, 1957) suggested the active urinary pre-carcinogen might be 2 - amino - 1 - napthylglucuronide which, when hydrolysed in urine by β-glucuronidase, could release 3,2-amino-1-naphthol, which they postulated was the ultimate reactive carcinogen. This suggestion was discounted at the time, partly because both 2-amino-1-naphthylglucuronide and β-glucuronidase were present in rat urine, yet, as everyone knew, rats "do not develop bladder cancer when dosed with 2-naphthylamine" (Deichmann & Radomski, 1963).

(a)

(b)

FIGS. 10(a), (b).—The luminal face of cells from an area of poorly differentiated epithelium on the surface of the tumour shown in Fig. 6. The cells are relatively undifferentiated, have none of the markers of normal urothelial superficial cells and are covered with numerous microvilli. At higher magnification the microvilli are seen to be covered by a fine filamentous glycocalyx which is not seen in association with the differentiated luminal membrane in normal bladders. Electron micrographs. (a) ×4000; (b) ×30,000.

After the demonstration by the Millers and their colleagues that N-hydroxy-2-acetylaminofluorene is a prominent metabolite of another arylamine carcinogen, namely 2-acetylaminofluorene (Cramer *et al.*, 1960; Miller *et al.*, 1961), Troll and his co-workers demonstrated the presence of N-hydroxy-2-naphthylamine in both human and dog urine (Troll & Nelson, 1961). This compound, unlike the parent arylamine, was carcinogenic when introduced directly into the bladder lumen of mice and dogs (Boyland *et al.*, 1964; Clayson & Cooper, 1970; Radomski *et al.*, 1971). Radomski *et al.* (1973*a*) were the first to suggest that the glucuronic acid conjugate of the N-hydroxy metabolite was the important carcinogenic urinary metabolite in the dog and they also detected *N*-hydroxy-2-naphthylamine in the urine of monkeys (Radomski *et al.*, 1973*b*). Recently, small but significant quantities (0·3% of the dose) of this compound and its *N*-glucuronide have

FIG. 11.—P2 papillary plus nodular transitional-cell carcinoma at the base of the ureter in a 2-naphthylamine-treated rat. Neoplastic urothelium (arrow) from the ureter has penetrated the muscle sheath separating the ureter from the bladder. H. & E. stained wax section. × 70.

been detected in rats given 300 mg 2-naphthylamine/kg body wt per week (Kadlubar *et al.*, 1978). Futhermore, 2-naphthylamine has been shown to be metabolized in the rat as in the dog, through N-oxidation by mixed-function oxidases in the liver, followed by N-glucuronidation by hepatic glucuronyl transferases (Kadlubar *et al.*, 1977). The N-hydroxyglucuronides are excreted *via* the urine where there is a non-enzymic, pH-dependent release of the free, carcinogenic N-hydroxy-2-naphthylamine from its glucuronide conjugate. Relatively more (47%) of the free *N*-hydroxy derivative is found if the urine of the treated rats is made acidic (pH $5{\cdot}7 \pm 0{\cdot}3$) than if it is made alkaline (pH $7{\cdot}7 \pm 0{\cdot}2$), when only 29% is in the form of the free N-hydroxy compound (Kadlubar *et al.*, 1978). These authors suggested that urinary pH is the controlling factor in 2-naphthylamine-induced bladder cancer and that the greater acidity of dog and human urine (pH 5·0–6·0), by comparison with that of the rat which they quote as pH 6·4–6·7, accelerates the rate of hydrolysis of the glucuronide conjugate, thus accounting for the greater susceptibility of man and dog to 2-naphthylamine carcinogenesis. If this is true, the variable results obtained with the rat in different laboratories could be explained not only by variations in the strain of rat but also by different diets which can affect urinary pH. For example, the urinary pH of 6·0–6·5 of female Wistars maintained on Dixon's 41B diet in this laboratory (Chowaniec & Hicks, 1979) is slightly lower than that found by Kadlubar and his colleagues.

By comparison with the relatively low doses of MNU, BBN and FANFT required to produce bladder cancer in the rat, a very high dose of 2-naphthylamine was used here to produce less than a 100% incidence. This suggests that 2-naphthylamine is a very weak carcinogen for the rat. However, if the amounts of the carcinogenic urinary metabolite are calculated from the data of Kadlubar *et al.* (1978) at a urinary pH of 6·0–6·5, only about 0·26% (0·78 mg) of the dose of 2-naphthylamine is likely to appear as an N-hydroxylation product in the urine and of that only about 40%, namely 0·3 mg, as free N-hydroxy-2-naphthylamine. The rats used in this study would have received an effective dose of only $\sim$0·3 mg/kg/week of this compound, equivalent to a total dose of less than 10 mg/animal/year. The rat urothelium may thus be sensitive to this metabolite of 2-naphthylamine, but the species or individuals within the species may be protected from its formation by a relatively high urinary pH.

Attractive though this proposition may be, it is not the only possible metabolic pathway for 2-naphthylamine in the bladder. Miyakawa *et al.* (1973) used glucosaccharo-(1,4)(6,3)-dilactone to obtain a 97% inhibition of urinary $\beta$-glucuronidase activity and at the same time reduced from 18 to 5% the incidence of bladder cancer in 2-acetylaminofluorene-treated rats maintained on a vitamin $B_6$-deficient diet. $\beta$-glucuronidase is also active in the lysosomes of the bladder superficial cells (Kanczack *et al.*, 1965). When the bladder contracts, small samples of urine are invaginated in fusiform vacuoles which are subsequently engulfed and digested by the lysosomes, thus exposing any N-hydroxyglucuronide present to the action of the lysosomal $\beta$-glucuronidase (Hicks, 1966, 1975). The active carcinogenic metabolite 2-naphthylamine may thus be released within the cells, irrespective of any pH-dependent non-enzymic mechanism in the urine. Depending on the relative importance of these 2 mechanisms, differences in species susceptibility may depend as much on the relative activities of tissue-bound $\beta$-glucuronidase in different species as on the pH of the urine.

The widely reported failure of the 2-naphthylamine-treated rat (and rabbit) to develop bladder cancer has been something of an anomaly for many years, and has also been largely responsible for a widespread belief that the rat is an inappropriate model for studies relating to human bladder cancer. The early experimental studies with dogs and rodents were technically difficult, time-consuming and expensive and done at a time when available biochemical analytical techniques were too insensitive to detect every metabolite of 2-naphthylamine. It is more remarkable that so much of the excellent pioneering work done in the 1950s remains valid today, than it is surprising that an occasional misinterpretation relating to species' susceptibility should have been made. However, in the light of recent metabolic studies and this report of a positive biological response of the female Wistar rat to oral doses of 2-naphthylamine, it should now be recognized that the rat is not completely resistant to the carcinogenic effect of 2-naphthylamine and that in the right experimental conditions rats, like dog and man, may develop bladder cancer.

This work was supported by a generous grant from the Cancer Research Campaign.

### REFERENCES

BONSER, G. M. (1943) Epithelial tumours of the bladder in dogs induced by pure $\beta$-naphthylamine. *J. Pathol. Bacteriol.*, **55**, 1.

BONSER, G. M., CLAYSON, D. B., JULL, J. W. & PYRAH, L. N. (1952) The carcinogenic properties of 2-amino-1-naphthol hydrochloride and its parent amine 2-naphthylamine. *Br. J. Cancer*, **6**, 412.

BONSER, G. M., CRABBE, J. G. S., JULL, J. W. & PYRAH, L. N. (1954) Induction of epithelial neoplasms in urinary bladder of dog by intravesical injection of chemical carcinogen. *J. Pathol. Bacteriol.*, **68**, 561.

BONSER, G. M., CLAYSON, D. B., JULL, J. W. & PYRAH, L. N. (1956) The carcinogenic activity of 2-naphthylamine. *Br. J. Cancer*, **10**, 533.

BOYLAND, E., BUSBY, E. R., DUKES, C. E., GROVER, P. L. & MANSON, D. (1964) Further experiments on implantation of materials into the urinary bladder of mice. *Br. J. Cancer*, **18**, 575.

BOYLAND, E., MANSON, D. & ORR, S. F. D. (1957) The biochemistry of aromatic amines. 2. The conversion of arylamines into arylsulphonic acids and arylamine-N-glucosiduronic acids. *Biochem. J.*, **65**, 417.

CASE, R. A. M., HOSKER, M. E., McDONALD, D. B. & PEARSON, J. T. (1954) Tumours of the urinary bladder in workmen engaged in the manufacture and use of certain dyestuff intermediates in the British chemical industry. I. The role of aniline, benzidine, alpha-naphthylamine and beta-naphthylamine. *Br. J. Industr. Med.*, **11**, 75.

CHOWANIEC, J. & HICKS, R. M. (1979) The response of the rat to saccharin with particular reference to the urinary bladder. *Br. J. Cancer*, **39**, 355.

CLARKE, H. E., COATES, M. E., EVA, J. K. & 5 others (1977) Dietary standards for laboratory animals. *Lab. Anim.*, **11**, 1.

CLAYSON, D. B. & COOPER, E. H. (1970) Cancer of the urinary tract. *Adv. Cancer Res.*, **13**, 271.

CONZELMAN, G. M. & MOULTON, J. E. (1972) Dose-response relationships of the bladder tumorigen 2-naphthylamine: a study in beagle dogs. *J. Natl Cancer Inst.*, **49**, 193.

CONZELMAN, G. M., JR, MOULTON, J. E., FLANDERS, L. E., SPRINGER, K. & CROUT, D. W. (1969) Induction of transitional cell carcinomas of the urinary bladder in monkeys fed 2-naphthylamine. *J. Natl Cancer Inst.*, **42**, 825.

CRAMER, J. W., MILLER, J. A. & MILLER, E. C. (1960) N-hydroxylation: A new metabolic reaction observed in the rat with the carcinogen 2-acetylaminofluorene. *J. Biol. Chem.*, **235**, 885.

DEICHMANN, W. B. & RADOMSKI, J. L. (1963) The carcinogenicity and metabolism of 2-naphthylamine and related compounds. *Ind. Med. Surg.*, **32**, 161.

DEICHMANN, W. B. & RADOMSKI, J. L. (1969) Carcinogenicity and metabolism of aromatic amines in the dog. *J. Natl Cancer Inst.*, **43**, 263.

ERTÜRK, E., PRICE, J. M., MORRIS, J. E. & 4 others (1967) The production of carcinoma of the bladder in rats by feeding N(4-(5-nitro-2-furyl)-2-thiazolyl) formamide. *Cancer Res.*, **27**, 1998.

FRIEDELL, G. H., JACOBS, J. B., NAGY, G. K. & COHEN, S. M. (1977) The pathogenesis of bladder cancer. *Am. J. Pathol.*, **89**, 431.

FUKUSHIMA, S., HIROSE, M., TSUDA, H. & 4 others (1976) Histological classification of urinary bladder cancers in rats induced by N-butyl-N-(4-hydroxy-butyl) nitrosamine. *Gann*, **67**, 81.

HADIDIAN, Z., FREDRICKSON, T. N., WEISBURGER, E. K., WEISBURGER, J. H., GLASS, R. M. & MANTEL, N. (1968) Tests for chemical carcinogens. Report on the activity of derivatives of aromatic amines, nitrosamines, quinolines, nitroalkanes, amides, epoxides, aziridines and purine anti-metabolites. *J. Natl Cancer Inst.*, **41**, 985.

HALDANE, J. B. S. (1957) Aunt Jobisca, the Bellman, and the Hermit, In *The Rationalist Annual* (Ed. H. Hawton). London: Watts & Co. p. 15.

HICKS, R. M. (1966) The function of the Golgi complex in transitional epithelium. *J. Cell Biol.*, **30**, 623.

HICKS, R. M. (1968) Hyperplasia and cornification of the transitional epithelium in the vitamin A-deficient rat. *J. Ultrastruct. Res.*, **22**, 206.

HICKS, R. M. (1969) Nature of the keratohyalin-like granules in hyperplastic and cornified areas of transitional epithelium in the vitamin A-deficient rat. *J. Anat.*, **104**, 327.

HICKS, R. M. (1975) The mammalian urinary bladder: an accommodating organ. *Biol. Rev.*, **50**, 215.

HICKS, R. M. (1980) Multistage carcinogenesis in the urinary bladder. *Br. Med. Bull.*, **36**, 39.

HICKS, R. M. & CHOWANIEC, J. (1978) Experimental induction, histology and ultrastructure of hyperplasia and neoplasia of the urinary bladder epithelium. *Int. Rev. Exp. Pathol.*, **18**, 199.

HICKS, R. M. & WAKEFIELD, J. ST J. (1972) Rapid induction of bladder cancer in rats with N-methyl-N-nitrosourea. 1. Histology. *Chem.-Biol. Interact.*, **5**, 139.

HICKS, R. M. & WAKEFIELD, J. ST J. (1976) Experimental urothelial tumours. In *The Scientific Foundation of Urology*, Vol. II (Ed. Innes Williams & Chisholm). London: Heinemann Medical Books Ltd. p. 302.

HUEPER, W. C., WILEY, F. H. & WOLFE, H. D. (1938) Experimental production of bladder tumors in dogs by administration of beta-naphthylamine. *J. Industr. Hyg.*, **20**, 46.

ITO, N. (1982) Effects of promoters on N-butyl-N-(4-hydroxybutyl)nitrosamine induced bladder carcinogenesis in the rat. In *Health Effects of Tumor Promotion* (Ed. Pereira) *Environ. Hlth. Persp.* **50** (in press).

ITO, N., HIASA, Y., TAMAI, A., OKAJIMA, E. & KITAMURA, H. (1969) Histogenesis of urinary bladder tumours induced by N-butyl-N-(4-hydroxybutyl)nitrosamine in rats. *Gann*, **60**, 401.

JACOBS, J. B., ARAI, M., COHEN, S. M. & FRIEDELL, G. H. (1977) A long-term study of reversible and progressive urinary bladder cancer lesions in rats fed N-(4-(5-nitro-2-furyl)-2-thiazolyl)formamide. *Cancer Res.*, **37**, 2817.

KADLUBAR, F. F., MILLER, J. A. & MILLER, E. C. (1977) Hepatic microsomal N-glucuronidation and nucleic acid binding of N-hydroxy arylamines in relation to urinary bladder carcinogenesis. *Cancer Res.*, **37**, 805.

KADLUBAR, F. F., FLAMMANG, T. & UNRUH, L. (1978) The role of N-hydroxy arylamine N-glucuronides in arylamine-induced urinary bladder carcinogenesis: Metabolite profiles in acidic, neutral and alkaline urines of 2-naphthylamine and 2-nitronaphthalene-treated rats. In *Conjugation Reaction in Drug Biotransformation* (Ed. Aitio). New York: Elsevier/North-Holland Press. p. 443.

KANCZAK, N. M., KRALL, J. I., HAYES, E. R. & ELLIOTT, W. B. (1965) Lysosomal fractions from transitional epithelium. *J. Cell Biol.*, **24**, 259.

KOSS, L. G. (1979) Mapping of the urinary bladder: Its impact on the concepts of bladder cancer. *Hum. Pathol.*, **10**, 533.

KOSS, L. G., MELAMED, M. R. & KELLY, R. E. (1969) Further cytologic and histologic studies of bladder lesions in workers exposed to paraaminodiphenyl: Progress report. *J. Natl Cancer Inst.*, **43**, 233.

KUNZE, E. & SCHAUER, A. (1977) Morphology, classification and histogenesis of N-butyl-N-(4-hydroxybutyl)-nitrosamine-induced carcinomas in the urinary bladder of rats. *Z. Krebsforsch.*, **88**, 273.

LITTLEFIELD, N. A., GREENMAN, D. L., FARMER, J. H. & SHELDON, W. G. (1979) Effects of continuous and discontinued exposure to 2-AAF on urinary bladder hyperplasia and neoplasia. *J. Environ. Pathol. Toxicol.*, **3**, 35.

MELAMED, M. R., VOUTSA, N. G. & GRABSTALD, H.

(1964) Natural history and clinical behaviour of *in situ* carcinoma of the human urinary bladder. *Cancer*, **17**, 1533.

MILLER, E. C., MILLER, J. A. & HARTMANN, H. A. (1961) N-hydroxy-2-acetylaminofluorene: A metabolite of 2-acetylaminofluorene with increased carcinogenic activity in the rat. *Cancer Res.*, **21**, 815.

MIYAKAWA, M., YOSHIDA, O., HARADA, T. & KATO, T. (1973) The effect of urinary β-glucuronidase inhibitor on the induction of bladder tumors with 2-acetylaminofluorene in rats. *Invest. Urol.*. **10**, 256.

NAKANISHI, K., HAGIWARA. A., SHIBATA, M., IMALDA, K., TATEMATSU, M. & ITO, N. (1980) Dose response of saccharin in induction of urinary bladder hyperplasias in Fischer 344 rats pretreated with N-butyl-N-(4-hydroxybutyl)nitrosamine. *J. Natl Cancer Inst.*, **65**, 1005.

PURCHASE, I. F. H. (1980) Inter-species comparisons of carcinogenicity. *Br. J. Cancer*, **41**, 454.

RADOMSKI, J. L., BRILL, E., DIECHMANN, W. B. & GLASS, E. M. (1971) Carcinogenicity testing of N-hydroxy and other oxidation and decomposition products of 1- and 2-naphthylamine. *Cancer Res.*, **31**, 1461.

RADOMSKI, J. L., CONZELMAN, G. M. JR, REY, A. A. & BRILL, E. (1973b) N-Oxidation of certain aromatic amines, acetamides, and nitro compounds by monkeys and dogs. *J. Natl Cancer Inst.*, **50**, 989.

RADOMSKI, J. L., REY, A. A. & BRILL, E. (1973*a*) Conjugates of the N-hydroxy metabolites of aromatic amines in dog urine. *Proc. Am. Ass. Cancer Res.*, **14**, 126.

SAFFIOTTI, U., CEFIS, F., MONTESANO, R. & SELLAKUMAR, A. R. (1967) Induction of bladder cancer in hamsters fed aromatic amines. In *Bladder Cancer. A Symposium* (Eds Deichmann & Lampe) Birmingham, Alabama: Aesculapius. p. 129.

SELLAKUMAR, A. R., MONTESANO, R. & SAFFIOTTI, U. (1969) Aromatic amines carcinogenicity in hamsters. *Proc. Am. Ass. Cancer Res.*, **10**, 78.

TROLL, W. & NELSON, N. (1961) N-hydroxy-2-napthylamine, a urinary metabolite of 2-naphthylamine in man and dog. *Fedn. Proc.*, **20**, 41.

YOSHIDA, M., NUMOTO, S. & OTSUKA, H. (1979) Histopathological changes induced in the urinary bladder and liver of female BALB/c mice treated simultaneously with 2-naphthylamine and cyclophosphamide. *Gann*, **70**, 645.